# Pharmacotherapeutic Options in Drug-Resistant Bipolar Depression: From Molecular Mechanisms to Rational Polypharmacotherapy

**DOI:** 10.3390/biomedicines14061185

**Published:** 2026-05-23

**Authors:** Dominik Jucha, Michał Klimas, Dominika Wiśniewska, Martyna Winiarska, Mateusz Szczupak, Jacek Kobak, Sabina Krupa-Nurcek

**Affiliations:** 1 Faculty of Medicine, Collegium Medicum, University of Rzeszów, 35-310 Rzeszów, Poland; dominik.jucha125@gmail.com (D.J.); michal.klimas02@gmail.com (M.K.); dominika.wisniewska.001@gmail.com (D.W.); martynawiniarska44@gmail.com (M.W.); 2 Department of Anaesthesiology and Intensive Therapy of the Nicolaus Copernicus Hospital in Gdańsk, 80-808 Gdansk, Poland; szczupak.mateusz@icloud.com; 3 Department of Otolaryngology, Faculty of Medicine, Medical University of Gdańsk, 80-210 Gdansk, Poland; 4 Department of Surgery, Faculty of Medicine, Collegium Medicum, University of Rzeszów, 35-310 Rzeszów, Poland; sabinakrupa@o2.pl

**Keywords:** bipolar disorder, drug-resistant bipolar depression, TRBD, polypharmacotherapy, augmentation, second-generation antipsychotics, SSRIs, normothymic drugs, ketamine, pramipexole

## Abstract

**Background/Objectives**: Bipolar disorder affects about 40 million people worldwide, and the greatest burden of the disease is associated with depressive episodes. About 25% of patients experience drug-resistant depression, in which standard treatment turns out to be insufficient, and monotherapy often does not bring full remission. Despite the use of second-generation antipsychotics, the effectiveness of therapy in TRBD remains limited, which necessitates rational polypharmacotherapy and augmentation strategies. The paper discusses the receptor mechanisms of drug combination, current therapeutic regimens and new interventions such as ketamine acting on the glutamate anergic system. The aim was to synthetically compare the efficacy and safety of available augmentation strategies and polypharmacotherapy. **Methods**: The material consists of published clinical, observational and randomized trials on pharmacotherapy of drug-resistant bipolar depression, including atypical neuroleptics, ketamine, pramipexole, modafinil, lamotrigine, celecoxib and memantine. The authors analyze receptor mechanisms, neurobiological data and clinical trial results, comparing them with current definitions of TRBD according to ISBD and CINP. Biomarker data, such as the Systemic Immune-Inflammation Index, and the results of neuroimaging and metabolomic studies were also used in the work. **Results:** The analysis showed that atypical neuroleptics showed limited efficacy and high rates of side effects, while ketamine has the fastest and most pronounced antidepressant effect with a low risk of phase change. Pramipexole has shown promise in terms of long-term efficacy, but its use reduces the high risk of induction of mania and impulse control disorders. Celecoxib as an anti-inflammatory therapy significantly increased response and remission rates compared to escitalopram alone, and memantine showed only an early, short-term antidepressant effect. The results highlight that TRBD requires targeted polypharmacotherapy, with the most promising directions being glutamatergic modulation and anti-inflammatory therapies. **Conclusions**: Drug-resistant bipolar depression requires a departure from classical monotherapy in favor of rational, mechanistically justified polypharmacotherapy, targeting complex monoaminergic, glutamatergic and neuroinflammatory disorders. Available data indicate that ketamine has the greatest clinical potential among the current strategies, characterized by a rapid onset of action and a favorable safety profile compared to atypical neuroleptics or dopamine agonists. Modulation of inflammatory processes with the use of celecoxib also has promising results, which highlights the importance of biomarkers and personalization of therapy. However, further, large, and well-designed studies are needed to unambiguously determine optimal treatment strategies for TRBD and to verify the effectiveness of new pharmacological interventions.

## 1. Introduction

Bipolar disorder is characterized by chronic periods of mania or hypomania, euthymia, and depression. Depressive episodes predominate in most cases, and the low mood phase—due to its prolonged duration and frequent coexistence of mixed features and anxiety—is most responsible for profound impairments in patients’ psychosocial functioning. Furthermore, the risk of suicide attempts and completed suicides in bipolar depression remains particularly high, highlighting the importance of this phase of the illness in clinical practice [1].

Treatment-resistant bipolar depression is emerging as a distinct, complex clinical problem. TRBD is not merely a phenomenon of poor treatment response, but a condition requiring its own conceptualization, particularly in the context of clinical trials and patient qualification for subsequent stages of therapy. Due to the limited effectiveness of some available therapeutic strategies and definitional difficulties, this area remains one of the greatest challenges in contemporary psychiatry [1,2]. According to the latest consensus statement from the International Society of Bipolar Disorders (ISBD), treatment-resistant bipolar depression is defined as the failure to achieve sustained symptomatic remission after at least two consecutive, adequate pharmacological treatment trials, with separate criteria for the selection of medications, for both bipolar I and bipolar II disorder. To be considered adequate, a treatment trial must employ a clinically proven effective medication at an appropriate dose and for an appropriate duration (at least 6 or 8 weeks, depending on the medication used, as indicated in Table 1), while maintaining adequate patient compliance. A key aspect from the perspective of research methodology and clinical practice is clearly distinguishing between inadequate response to treatment (insufficient response) and TRBD. Inadequate response refers to a situation in which a patient fails to achieve full remission or demonstrates only partial improvement after a single therapeutic intervention, or when treatment fails to meet strictly defined criteria for appropriateness, for example, due to too low a dose, too short a treatment duration, treatment intolerance, or poor adherence. Diagnosis of full TRBD requires documented failure of at least two properly conducted treatment lines, designated separately for type I and type II of the disease [2]. Crucially, these two failed trials do not need to involve medications from different pharmacological classes; for example, failure to respond to two different atypical antipsychotics is sufficient for a TRBD diagnosis. Medications used in the treatment of a depressive episode of bipolar disorder are presented in Table 1.

Compliance with the above treatment regimens, taking into account the appropriate dose and duration of therapy, allows for a more precise separation of the population of patients suffering from TRBD, which is the starting point for the safe and justified implementation of advanced algorithms. In addition, it is necessary to understand the receptor mechanism of the drugs used as well as the intricate nature of the disease.

## 2. Materials and Methods

This review was conducted to synthesize current evidence on pharmacotherapeutic strategies for treatment-resistant bipolar depression (TRBD), integrating data from clinical trials, observational studies, mechanistic research, and biomarker analyses. The source material consisted of published clinical, observational, and randomized controlled studies evaluating pharmacological interventions in TRBD, including atypical antipsychotics, ketamine, pramipexole, modafinil, lamotrigine, celecoxib, and memantine, as stated in the manuscript content.

### 2.1. Literature Search Strategy

A comprehensive literature search was performed in major biomedical databases (PubMed, Scopus, Web of Science). Searches included combinations of the following keywords: bipolar depression, treatment-resistant bipolar depression, TRBD, augmentation, polypharmacotherapy, ketamine, pramipexole, modafinil, lamotrigine, celecoxib, memantine, second-generation antipsychotics, and glutamatergic modulation. No date limits were applied to ensure inclusion of both foundational and recent studies. Only articles published in English and available in full text were considered.

### 2.2. Eligibility Criteria

Studies were included if they met the following criteria: involved adult patients diagnosed with bipolar disorder (BD-I or BD-II); evaluated pharmacological treatment of depressive episodes meeting criteria for inadequate response or TRBD, consistent with ISBD and CINP definitions; reported clinical outcomes of augmentation therapies such as response, remission, symptom reduction, tolerability, or biomarker changes; and were randomized controlled trials, open-label trials, retrospective or prospective observational studies, or mechanistic human studies.

Exclusion criteria included: studies focusing exclusively on unipolar depression; preclinical or animal studies unless directly informing mechanistic interpretation; case reports unless illustrating clinically relevant phenomena not captured in larger studies.

### 2.3. Data Extraction and Synthesis

Data were extracted independently by the authors and included study design, sample characteristics, diagnostic criteria, definitions of treatment resistance, intervention details, outcome measures, and safety profiles. Mechanistic findings (e.g., receptor pharmacodynamics, neuroimaging correlates, inflammatory biomarkers, metabolomic signatures) were integrated to contextualize clinical outcomes, as described in the manuscript’s methodological description. Given the heterogeneity of study designs, interventions, and outcome measures, a quantitative meta-analysis was not feasible. Therefore, a qualitative, integrative synthesis was performed, emphasizing: convergence of evidence across pharmacological classes, mechanistic plausibility of treatment strategies, comparative efficacy and tolerability, alignment with contemporary TRBD definitions.

### 2.4. Definitions and Conceptual Framework

The review adhered to the most recent ISBD and CINP criteria for defining adequate treatment trials and TRBD, including dose, duration, and treatment-class requirements. These definitions were used to interpret study populations and classify interventions within a consistent clinical framework, as outlined in the manuscript’s introductory methodological section.

## 3. Results

### 3.1. Pathophysiological Theories of Bipolar Disorder

*Monoaminergic hypothesis*

The pathophysiology of bipolar disorder is extremely complex. First, it is necessary to discuss abnormalities in monoaminergic neurotransmission within the cortico-limbic circuits responsible for the regulation of affect, drive, and reward. Within the serotonergic system, the 5-HT1A receptor and the serotonin transporter (5-HTT) are particularly important; decreased binding of postsynaptic 5-HT1A receptors, particularly in the mesiotemporal cortex, has been reported in bipolar depression. Analyses have shown that these abnormalities may be particularly severe in women, and an inverse correlation between receptor density and cortisol concentration has been observed, linking brain biochemistry with the hormonal response to chronic stress [3]. Imaging and genetic data also indicate the involvement of tryptophan hydroxylase 2 (TPH2) and 5-HT2A and 5-HT4 receptors, and these abnormalities have been localized, among others, in the hippocampus, amygdala, thalamus, insula, anterior cingulate cortex (ACC) and medial prefrontal cortex [4].

D2/D3 receptors, the dopamine transporter (DAT), and the mesolimbic and mesocortical projection fibers of the dopaminergic pathway are crucial in the dopaminergic system. The manic phase may be associated with increased availability of D2/3 receptors and hyperreactivity of the reward system. Imaging studies suggest an increased density and availability of D2/3 dopamine receptors in the striatum and increased activity of the reward system [5]. Additional review data indicate that the DAT dopamine transporter gene and D2 and D4 dopamine receptors have been implicated in bipolar disorder, and dopaminergic disorders should be considered in the context of broader dysregulation of cortico-limbic and striatal circuits [6]. Imaging studies indicate elevated DAT levels, which are associated with faster removal of the neurotransmitter from the synaptic cleft, which attenuates dopaminergic signaling and leads to the development of depressive symptoms [5]. In the noradrenergic system, disturbances in noradrenaline turnover are of particular importance. Of particular importance here is 3-methoxy-4-hydroxyphenylglycol (MHPG), the main metabolite of noradrenaline, whose levels correlate with the current phase of the illness and may reflect the severity of manic symptoms or the transition between mania and remission [7]. Attention is also paid to the involvement of the noradrenaline transporter (NET) and the presynaptic α2 receptor (autoreceptor), as increased noradrenergic transmission may alleviate the symptoms of bipolar depression but simultaneously increase the risk of switching to mania [8].

*Cortico-limbic circuits*

In addition to purely neurotransmitter theories, the model of dysregulation of cortico-limbic circuits, particularly the ventral prefrontal cortex-amygdala neural network responsible for the regulation of emotions, reward, and behavior, plays an important role in the pathophysiology of bipolar disorder. In this view, the ventral prefrontal cortex (VPFC) includes the orbitofrontal cortex, the inferior and rostral parts of the frontal lobe, and the ventral and pregenual parts of the cingulate gyrus. This structure serves as a superior control over emotional and motivational responses, while the amygdala is responsible for the rapid processing of the emotional significance of stimuli. Under normal conditions, the VPFC exerts an inhibitory influence on the activity of the amygdala, stabilizing affective responses and enabling an adequate assessment of the consequences of behavior. In bipolar disorder, this balance is disturbed: impaired VPFC function and simultaneous amygdala hyperreactivity lead to a loss of affective homeostasis, which may explain impulsivity, increased pursuit of reward and risk in mania, as well as impaired emotion processing in depression. This system does not operate in isolation but is interconnected with the hippocampus, ventral striatum, thalamus, and hypothalamus. The hippocampus may be responsible for the memory and cognitive components of the disorder, the ventral striatum for motivation, drive, and reward processing, and the hypothalamus for neurovegetative symptoms such as sleep, appetite, and sexuality [9].

*Glutamatergic hypothesis*

The glutamatergic theory posits that dysregulation of excitatory glutamatergic transmission, involving both ionotropic and metabotropic receptors, plays a significant role in BD. NMDA and AMPA receptors, as well as genes associated with their function, such as GRIN1, GRIN2A, GRIN2B, GRM3, and GRM4, are most frequently implicated. Of particular importance are the hippocampus, prefrontal cortex, and anterior cingulate cortex, where glutamatergic receptor expression and synaptic transmission abnormalities have been reported [10]. Postmortem studies have demonstrated, among other things, a decrease in the expression of the NR1 and NR2A subunits of the NMDA receptor in the hippocampus and an increase in the expression of the vesicular glutamate transporter 1 (VGluT1) and markers of synaptic plasticity in the anterior cingulate gyrus, suggesting a simultaneous impairment of the postsynaptic response to glutamate and presynaptic neurotransmitter release. The observed postsynaptic reduction in NMDA receptor density is most likely a compensatory mechanism, as the key role in the pathogenesis of damage is attributed not to physiological synapses, but to disturbances in glial-neuronal interactions. Under the influence of inflammatory mediators, phenotypically altered astrocytes lose the ability to remove excess neurotransmitter, instead releasing abnormal amounts of glutamate into the extrasynaptic space [6,11]. This phenomenon is exacerbated by activated microglia, which, through increased activity of the enzyme indoleamine 2,3-dioxygenase, increase the production of quinolinic acid (QA), an extremely potent, neurotoxic NMDA receptor agonist. The net result of these processes is toxic hyperstimulation of the extrasynaptic pool of NMDA receptors, which induces suppression of brain-derived neurotrophic factor (BDNF) synthesis and directly activates intracellular proapoptotic cascades, leading to cell death. This model explains well how excessive activation of the glutamatergic system can promote excitotoxicity, impaired neuroplasticity, and the perpetuation of disease episodes [6]. From a clinical perspective, the glutamatergic theory is important because it links neurotransmission disorders with mechanisms of synaptic plasticity and provides a biological rationale for therapies that go beyond monoamine modulation alone [10]. Disturbances in neuroplasticity and intracellular signaling constitute one of the key biological models of bipolar disorder. Particularly important is the decreased activity of neurotrophic systems, especially those dependent on BDNF and the TrkB receptor, which may lead to impaired synaptic plasticity, reduced neuronal resistance to stress, and worsening adaptive processes during subsequent disease episodes [12]. In parallel, dysregulation of intracellular signaling pathways, particularly GSK-3β and the Wnt/β-catenin pathway, which participate in the regulation of neurogenesis, cell survival, inflammatory response, and synaptic remodeling, has been described. Excessive GSK-3β activity may promote the intensification of proapoptotic processes, reduced expression of neurotrophic factors, and the perpetuation of neuroprogression [13]. Protein kinase C (PKC), particularly present in frontolimbic structures such as the prefrontal cortex, hippocampus, and amygdala, also plays a significant role. PKC influences neuronal excitability, neurotransmitter release, receptor regulation, and long-term synaptic remodeling processes; therefore, its dysregulation may be an important link between neurotransmission disorders and the perpetuation of affective symptoms and increasing drug resistance [14].

*Inflammatory hypothesis*

The inflammatory theory and the concept of neuroprogression conceptualize BD as a disease in which subsequent affective episodes lead to the gradual perpetuation of biological changes, not merely to transient mood disturbances. Chronic activation of the immune system, involving microglia and astroglia, and increased levels of proinflammatory cytokines, particularly IL-1β, IL-6, and TNF-α, as well as activation of the NF-κB pathway, play a significant role here, disrupting neuroplasticity and synaptic transmission [15]. Simultaneously, oxidative stress, disturbances in calcium homeostasis, and mitochondrial dysfunction occur, resulting in decreased ATP synthesis, activation of apoptotic pathways, and decreased ability of nerve cells to regenerate [16]. In this perspective, neuroprogression means the increasing changes in neuronal networks with the duration of the disease and the number of episodes, which is clinically associated with shortened remission, deterioration of cognitive functions and a greater risk of drug resistance, including the development of TRBD [17].

#### 3.1.1. Definitional Foundations and Biological Theories of TRBD

The pathophysiological models described above allow for a better understanding of why, in some patients, bipolar depression may cease to respond to standard treatment over time. With recurring episodes, persistent disturbances in neuroplasticity, intracellular signaling, inflammatory response, cellular metabolism, and corticolimbic network integration become increasingly important, which may lead to a gradual decline in responsiveness to traditional pharmacological interventions. This understanding of drug resistance is consistent with the approach that views TRBD as a distinct and more complex clinical problem, rather than simply the ineffectiveness of a single drug [18]. A similar position is taken by the CINP guidelines, which emphasize that drug resistance in bipolar disorder should be defined operationally and considered separately for individual phases of bipolar disorder; in relation to a depressive episode, this means a lack of satisfactory response despite treatment consistent with current recommendations, administered at appropriate doses and for a sufficient duration, including both monotherapy and combination therapies [18]. In the Hidalgo-Mazzei et al. consensus definition, TRBD means the failure to achieve sustained symptomatic remission for eight consecutive weeks despite two adequate treatment trials, including at least two recommended monotherapies or one monotherapy and one combination therapy [19]. The more recent ISBD position statement emphasizes the need to standardize the definition of TRBD for clinical trials and practice, which reflects the growing importance of this phenotype as a distinct therapeutic problem and introduces a definition according to which the diagnosis of TRBD requires fulfillment of the criteria for the use of appropriate pharmacological options, taking into account the type of drug used, dose, duration, and ineffectiveness of two therapies [2].

At the biological level, increasing attention is being paid to inflammatory and immunometabolic markers. In an open-label biomarker study involving 52 patients with TRBD and 32 healthy controls, depression severity was assessed using the HAMD-17 scale. Patients were eligible for the study if they had a score of at least 18 points. Treatment response was defined as a score decrease of ≥50%, and remission as a score of ≤7 points after 8 weeks. The systemic immune-inflammation index (SII), calculated as the product of platelet and neutrophil counts divided by the lymphocyte count, did not differentiate patients with TRBD from healthy individuals and did not correlate independently with baseline depression severity. However, a higher baseline SII predicted a poorer treatment response in older patients. Importantly, a similar prognostic effect was observed for baseline neutrophil counts. Furthermore, higher SII was associated with lower baseline VEGF levels and higher IL-1β and CRP levels after 8 weeks of treatment, supporting the hypothesis of an increased inflammatory and immunometabolic burden in TRBD. The escitalopram plus celecoxib group also demonstrated better clinical outcomes than the escitalopram plus placebo group, further reinforcing the importance of the inflammatory pathway as a potential therapeutic target [20].

Emerging data suggest biological differences in TRBD in neuroimaging and metabolism. A study using ketamine demonstrated a rapid reduction in anhedonia in patients with TRBD, and this improvement was associated with increased glucose metabolism in the dorsal anterior cingulate cortex and putamen, suggesting the involvement of reward, motivation, and affect regulation circuits in the biology of drug resistance [21]. A pilot metabolomic analysis showed that the response to ketamine was associated with differences in lipid profiles and pathways related to mitochondrial function, suggesting the involvement of bioenergetic disturbances and cell membrane metabolism in the pathophysiology of TRBD [22].

#### 3.1.2. Pharmacotherapy of BD

The foundational treatment for acute bipolar depression typically begins with standard monotherapy utilizing agents approved by major regulatory agencies (FDA, EMA). For Bipolar I depression, first-line options primarily include atypical antipsychotics (quetiapine, lurasidone, cariprazine, lumateperone) and the olanzapine-fluoxetine combination, whereas for Bipolar II, approved options are limited to quetiapine and lumateperone; however, traditional mood stabilizers like lithium and lamotrigine remain widely used in everyday clinical practice. When initial monotherapy yields an insufficient response, the clinical pathway advances to polypharmacy. A common evidence-based approach involves combining mood stabilizers, such as augmenting lithium with lamotrigine, or adding adjunctive agents like modafinil, pramipexole, or levothyroxine. Traditional antidepressants are not recommended as monotherapy; their use is restricted to carefully selected cases and strictly requires co-administration with a mood stabilizer to prevent a manic switch. Ultimately, patients who fail to achieve sustained remission after at least two consecutive, adequately dosed standard treatments are diagnosed with treatment-resistant bipolar depression (TRBD) [1].

#### 3.1.3. Pharmacotherapy Strategies of TRBD

TRBD typically requires more complex treatment than standard monotherapy, as therapeutic responses in bipolar disorder are often incomplete, particularly with respect to depressive symptoms. Properly implemented polypharmacy, however, does not simply involve increasing the number of medications but rather selecting medications with complementary mechanisms of action, ensuring that each component of therapy has a clearly defined clinical and pharmacodynamic function. This approach is particularly important in refractory cases, in which monotherapy rarely leads to complete remission [23]. In practice, this means combining medications that affect various levels of the pathophysiology of bipolar depression—from monoaminergic and glutamatergic systems to neuroplasticity mechanisms. At the same time, available data indicate that the number of well-validated therapeutic options for TRBD remains limited, and most studies focus on augmentation strategies and combination therapy, rather than effective monotherapy [24]. Therefore, the treatment of TRBD should be understood as a procedure aimed at overcoming the incomplete therapeutic response, which is particularly common in the depressive components of bipolar disorder. Available data indicate that in refractory cases, most of the studied strategies are augmentative or based on combination therapy, reflecting the limited effectiveness of simple monotherapy regimens [24]. At the same time, contemporary reviews emphasize that the number of well-validated therapeutic options for bipolar depression remains small, and recommendations from various guidelines are not entirely consistent, which reinforces the need for individualized treatment and therapy selection based on the dominant clinical and biological mechanisms [25,26,27]. Table 2 shows targeted adjunctive strategies for the management of TRBD.

#### 3.1.4. Atypical Antipsychotics

*Aripiprazole*

Aripiprazole is an atypical neuroleptic with a complex receptor profile, acting primarily as a partial agonist at dopamine D2 and D3 and serotonin 5-HT1A receptors, and also exhibiting affinity for D4, 5-HT2C, and 5-HT7 receptors, while also acting as a 5-HT2A antagonist [26]. Common side effects include akathisia, insomnia, anxiety, agitation, nausea, and other gastrointestinal symptoms, as well as mild extrapyramidal symptoms. Aripiprazole has a relatively favorable metabolic profile, with a low propensity for hyperprolactinemia and a generally lower risk of weight gain than some other atypical neuroleptics [27]. Aripiprazole has only limited, preliminary clinical support for TRBD. In the study by Ketter et al., the drug was used as an augmentation in 30 outpatients with bipolar disorder type I, II, and NOS (Not Otherwise Specified), assessed as part of the STEP-BD program, who remained depressed despite previous treatment; aripiprazole was administered for an average of 84 ± 69 days. Clinical response was achieved in 27% of patients, remission in 13%, and the mean final dose was 15.3 ± 11.2 mg per day, with a wide range of 2.5 to 40 mg. However, the study also showed clear limitations in tolerability and durability of treatment, as 47% of patients discontinued therapy, including 17% due to ineffectiveness and 20% due to adverse events. These data suggest a possible antidepressant effect, but rather moderate and obtained in a small, uncontrolled trial [28]. The chart review study by Kemp et al. included only 12 patients with TRBD type I, II, or NOS who were added to aripiprazole due to an acute episode of major depression. After 8 weeks, a response, defined as at least a 50% reduction in the MADRS (Montgomery-Åsberg Depression Rating Scale) score, was achieved in 4 of 12 patients, or approximately 33%. However, tolerability was the most important issue, as new akathisia developed in 5 of 12 patients. This finding is why aripiprazole in TRBD is sometimes described as a possible option rather than a well-established drug, as the efficacy signal is weak and the risk of akathisia is high [29]. Additional context is provided by the prospective, randomized, double-blind, placebo-controlled study by Quante et al. The study included 23 hospitalized patients with a depressive episode in the course of bipolar I or II disorder. However, this was not a strict TRBD population, as the study included patients with a non-drug-resistant depressive episode treated with mood stabilizers (lithium, valproate) and SSRIs (citalopram). After 6 weeks of treatment, aripiprazole did not demonstrate a statistically significant advantage over placebo in reducing depressive symptoms, despite improvement observed in both groups. Aripiprazole treatment was also associated with poorer tolerability, as 4 patients discontinued participation due to adverse events, including 2 due to akathisia [30].

*Risperidone*

Risperidone is an atypical neuroleptic with an antagonistic profile, with very high affinity for serotonin 5-HT2A receptors (binding is 20 times stronger than clozapine and 170 times stronger than haloperidol) and potent activity at dopamine D2, D3, and D4 receptors; it also shows significant affinity for alpha-adrenergic and histamine H1 receptors, while its activity at muscarinic cholinergic receptors is low, which distinguishes it from more cholinolytic atypical neuroleptics [31]. The most important adverse effects of risperidone are extrapyramidal symptoms, hyperprolactinemia, sedation, weight gain, and orthostatic hypotension associated with alpha-adrenergic blockade; the risk of EPS is lower than with classical neuroleptics, but still clinically significant, especially at higher doses [32]. In a study of 66 patients with bipolar depression refractory to adequate treatment with a mood stabilizer and at least one antidepressant, patients were randomized to receive lamotrigine, inositol, or risperidone for up to 16 weeks. The efficacy of risperidone in this population was limited: the recovery rate was only 4.6%, compared to 17.4% for inositol and 23.8% for lamotrigine [33]. Therefore, the results of this study do not indicate that risperidone is a particularly effective augmentation strategy for TRBD.

*Cariprazine*

Cariprazine is an atypical neuroleptic acting primarily as a partial agonist at dopamine D3 and D2 receptors, with a clear preference for D3, and a partial 5-HT1A agonist; it exhibits 5-HT2B antagonist activity and moderate affinity for 5-HT2A, moderate affinity for histamine and adrenergic receptors, and low cholinergic activity, resulting in a low anticholinergic profile [34,35]. The most common adverse effects of cariprazine are akathisia, extrapyramidal symptoms, nausea, and insomnia, while the risk of hyperprolactinemia, significant metabolic disturbances, sedation, and QTc prolongation appears to be rather low [36]. In clinical practice, akathisia (as with aripiprazole) remains the most important limitation of tolerability [35]. Currently, mainly observational data are available in TRBD. A retrospective, multicenter observational study, based on the analysis of medical records of patients admitted or referred to psychiatric facilities between June 2022 and February 2023, assessed the efficacy and tolerability of cariprazine as augmentation therapy in 51 patients with TRBD, defined as failure of at least two appropriate treatments with two classes of antidepressants and two classes of mood stabilizers, including atypical antipsychotics. All patients received a therapeutic dose of a mood stabilizer, most commonly lithium, and less frequently valproate, and at least one antidepressant (28 patients used an SSRI, six patients each used an SNRI, a multimodal medication, and a TCA). After 4 weeks of treatment, at a mean cariprazine dose of 1.7 mg daily, 23.5% of patients achieved response and 21.6% achieved remission, with an overall clinical benefit exceeding 45%. Alongside improvements in depression, cariprazine demonstrated a significant short-term reduction in anxiety symptoms, with mean Hamilton Anxiety Rating Scale (HAM-A) scores substantially decreasing from 26.63 at baseline to 15.66 by the end of the observation period. Treatment was generally well tolerated, although adverse events were observed in 70.6% of patients, most commonly inner restlessness, somnolence, akathisia, and tremor; however, these were mild to moderate. In total, 7.8% of patients withdrew from the study, primarily due to adverse events, and no clinically significant manic or hypomanic switch was observed [37]. In turn, a more recent long-term follow-up study published in 2024, lasting 24 weeks, included 30 patients with TRBD treated with cariprazine at a dose of 1.5–3 mg per day as an augmentation to previous mood stabilizer and/or antidepressant treatment. It showed that the improvement was most pronounced in the first 4 weeks, when the average reduction in the HDRS (Hamilton Depression Rating Scale) score was approximately 40%, and anxiety symptoms decreased by approximately 37% (as measured by the Hamilton Anxiety Rating Scale, HAM-A). Further follow-up to week 24 showed only a moderate additional improvement in depression, approximately 15%, with a very high treatment discontinuation rate of nearly 70%; the main reasons for treatment discontinuation were ineffectiveness, clinical worsening, adverse events, and isolated hypomanic switches [38].

*Lurasidone*

Lurasidone is an atypical neuroleptic with a favorable and characteristic receptor profile, demonstrating high affinity for dopamine D2 and serotonin 5-HT2A and 5-HT7 receptors, for which it acts as an antagonist, as well as partial agonism for 5-HT1A receptors; it also demonstrates moderate affinity for noradrenergic α2C receptors, with minimal affinity for histamine H1 and muscarinic M1 receptors, which distinguishes it from more sedating and anticholinergic atypical neuroleptics [39,40]. This receptor profile has been associated not only with antipsychotic activity but also with potential antidepressant, anxiolytic, and procognitive effects [39]. The most common adverse effects of lurasidone include akathisia, somnolence, nausea, insomnia, and tremor, while its effect on body weight, lipid metabolism, and glycemia is usually minor [41]. Compared with some other atypical antipsychotics, lurasidone also exhibits low anticholinergic activity and a relatively favorable metabolic profile, although the incidence of adverse events increases with increasing dose [42]. A retrospective, multicenter observational study assessed the efficacy and tolerability of lurasidone as an augmentation in 60 adult patients with TRBD. The study included patients diagnosed with bipolar disorder according to the DSM-5-TR, in the current depressive episode, and in whom treatment resistance was defined as an inadequate response to at least two classes of mood stabilizers, including atypical antipsychotics, and at least two classes of antidepressants. Each trial had to be considered adequate in terms of therapeutic dose and duration. All patients received lurasidone as an adjunct to ongoing pharmacotherapy, and symptom severity was assessed using the HAMD-17 scale, the HAM-A scale, and the YMRS scale. The mean starting dose of lurasidone was 32.9 mg/day, increasing to 46.7 mg/day during follow-up, and the mean final dose reached 51.2 mg/day; the study dose range was 18–111 mg/day. The study population was clinically severe: the mean baseline HAMD-17 score was 25.9 ± 4.3 points and the HAM-A score was 24.5 ± 7.1 points, corresponding to moderate to severe depressive symptoms. After 4 weeks of treatment, the mean HAMD-17 score decreased to 16.3 ± 6.9 points, and clinical response was defined as a score reduction of at least 50%. This condition was met in 20 of the 60 patients. Remission, defined as a HAMD-17 score below 7 points, was achieved in only 2 patients (3.3%). Alongside the improvement in depressive symptoms, patients also experienced a progressive and significant reduction in anxiety, with the mean HAM-A score decreasing to 18.0 ± 10.0 points by the end of the 4-week treatment. Adverse events were reported in 68.3% of patients; the most common were weight gain, tremor, nausea, sedation or somnolence, internal tension, muscle rigidity, and akathisia, all of which were mild to moderate in severity. These results suggest that lurasidone may provide significant symptomatic improvement in some patients with TRBD, but the rate of complete remission at short-term follow-up remained low [43].

### 3.2. Ketamine

Ketamine, synthesized in the early 1960s as a safer alternative to phencyclidine (PCP), initially gained recognition solely as an effective drug for dissociative analgesia and analgesia. A breakthrough came in the 1990s, when its rapid antidepressant properties were unexpectedly discovered, ultimately leading to FDA approval of (S)-ketamine as an antidepressant in 2019. Historically, its effects were thought to be primarily due to its high affinity for and antagonism of the N-methyl-D-aspartate (NMDA) receptor. However, contemporary research indicates that a much broader, multifaceted mechanism of action is responsible for this drug’s unique clinical effects. Currently, it is believed that the key role in ketamine’s antidepressant action is played not only by NMDA receptor blockade, but also by signaling mediated by AMPA receptors, brain-derived neurotrophic factor (BDNF), and the mTOR kinase pathway, involving the monoaminergic and cholinergic systems, sigma-1 receptors, and voltage-gated calcium channels (VGCCs). Furthermore, its anesthetic and analgesic properties result from modulation of the opioid and endocannabinoid systems and antagonism of HCN (Hyperpolarization-Activated Cyclic Nucleotide-Gated Channels). This remarkable wealth of targets makes the discovery of ketamine’s antidepressant properties one of the most important achievements in modern pharmacotherapy [44]. In psychiatry, its particular importance stems from the very rapid onset of antidepressant action, occurring within hours or days, rather than weeks as with classic antidepressants [45]. The most common adverse effects of ketamine include dissociative symptoms, transient increases in blood pressure, dizziness, nausea, and somnolence [46]. Studies on bipolar depression and TRBD have also reported the possibility of hypomania or mania, but available data suggest that the risk of switching remains relatively low. The authors of the systematic review calculated that the overall incidence of manic/hypomanic symptoms (the so-called affective switch) for intravenous ketamine across all analyzed studies was 2.4% (5 of 208 patients) [47].

A randomized, double-blind, placebo-controlled study conducted in 2012 involved a selected group of 15 adult patients with bipolar I and II disorder. To ensure a rigorous definition of treatment resistance, only patients with documented lack of improvement after at least one adequate trial of antidepressant pharmacotherapy and failure of at least 4 weeks of treatment with a mood stabilizer (lithium or valproate) at appropriate therapeutic concentrations were included in the study. The results of the study demonstrated a significant advantage of ketamine over placebo. A clinical response, defined as a reduction in symptom severity of at least 50%, was achieved by 79% of patients receiving a single infusion of ketamine (0.5 mg/kg), compared to 0% in the control group. A highly significant indicator is the fact that 36% of patients achieved complete remission of depressive symptoms just 40 min after intravenous administration. A statistically significant difference in symptom reduction in favor of ketamine compared to placebo persisted for 3 days after the infusion. Alongside the antidepressant effects, ketamine also produced a rapid and significant reduction in anxiety symptoms—as measured by the HAM-A and VAS-Anxiety scales—which was observable as early as 40 min post-infusion. The procedure was characterized by a favorable safety profile, devoid of serious adverse events. The most frequently reported side effects were mild dissociative symptoms, feelings of intoxication, dizziness, and transient cognitive impairment; however, these were highly transient and occurred almost exclusively around the 40th minute of infusion [48]. In 2025, a retrospective, single-center observational study assessed the efficacy and tolerability of intravenous infusions of racemic ketamine in 59 patients with treatment-resistant depression in the course of bipolar I and II disorder. The study enrolled adult patients with bipolar I or II disorder who were in a current depressive episode resistant to at least two antidepressant treatment trials in combination with antimanic therapy. This population was significantly polypharmacologically challenged: 93% of patients were taking mood stabilizers or anticonvulsants, 78% were taking atypical antipsychotics, 100% were taking antidepressants (SSRIs, SNRIs, SA-RIs), and 98% were also taking stimulants or benzodiazepines. The treatment protocol involved administration of ketamine at an average dose of 0.8 mg/kg body weight, initially twice weekly for the first three weeks, then once weekly or less frequently based on clinical judgment. The primary endpoint was the change in the MADRS total score over 4 weeks. The median MADRS score at baseline was 39 points, which decreased to 18 points after 4 weeks of treatment. The rapid effect of the intervention proved particularly clinically significant. Statistical analysis (Friedman test, *p* < 0.0001) demonstrated significant improvement starting from the second week of therapy, when a significant reduction in median scores for internal tension (from 5 points at baseline to 3–4 points in week 2), sleep disturbances (from 3 to 2 points), and suicidal ideation (from 3 to 2 points) was observed. The treatment was generally well-tolerated: the most common adverse events included mild to moderate dissociation, dizziness, transient cognitive impairment, occasional nausea, and increased blood pressure, observed primarily during the infusion. Crucially, given the risks of antidepressant therapy, no patient experienced a phase shift to a manic episode (switch) throughout the entire observation period, and no premature withdrawals were noted in the study. These results suggest that intravenous ketamine may be an effective and relatively well-tolerated option in TRBD; however, the lack of randomization, lack of a control group, short follow-up period, and highly heterogeneous background treatment limit the strength of the conclusions [49].

### 3.3. Pramipexole

Pramipexole is a dopamine receptor agonist, originally introduced for the treatment of Parkinson’s disease and later also for restless legs syndrome (RLS); in psychiatry, interest in it stems from its prodopaminergic effects, which may be important in bipolar depression, especially in cases with predominant anergy, anhedonia, and psychomotor retardation [50]. Pharmacologically, pramipexole belongs to the D2, D3, and D4 receptor agonists, with a clear preference for the D3 receptor, which is particularly important from the point of view of its potential antidepressant action and impact on the reward system [51]. Unlike atypical neuroleptics, it does not antagonize dopamine receptors but enhances dopaminergic transmission, which provides a biological justification for its use as an augmentation in treatment-resistant depression in the course of bipolar disorder [52]. The most important adverse effects include somnolence, sudden sleep attacks, and impulse control disorders [53]. In psychiatry, the risk of phase switching, agitation, and hypomania should also be considered, although in available studies on bipolar depression, the drug was generally assessed as relatively well tolerated [54]. In a multicenter, randomized, double-blind, placebo-controlled study (PAX-BD) conducted in 2025, the efficacy and tolerability of pramipexole were assessed in 39 patients with treatment-resistant depression in the course of bipolar I and II disorder. Resistance in this study was defined as lack of response, poor tolerability, or contraindications to at least two of the four recommended medications (quetiapine, olanzapine, lamotrigine, or lurasidone). The population was polypharmacologically challenged—all participants were taking at least one mood stabilizer (lithium, valproate, lamotrigine, carbamazepine), 56% were taking an antidepressant (fluoxetine), and 46% were taking an atypical antipsychotic. The protocol involved slow titration of pramipexole to a target dose of 2.5 mg/day, with the final mean dose achieved by patients being 2.18 mg/day. Regarding the primary endpoint, after 12 weeks of treatment, the reduction in depressive symptoms on the Quick Inventory of Depressive Symptomatology (QIDS-SR) scale was more than twice as high in the pramipexole group compared to placebo. Despite a medium to large effect size (Cohen’s d = 0.72), this difference did not reach statistical significance (*p* = 0.087), which the authors directly attribute to a lack of statistical power resulting from the premature termination of the study and recruitment due to the COVID-19 pandemic. However, in the longer follow-up period covering the time to study exit (maximum 48 weeks), pramipexole demonstrated a statistically significant advantage—46% of patients in the study group achieved a treatment response compared to 6% in the placebo group, and 31% of patients on pramipexole achieved complete remission compared to none in the control group (0%). Despite promising long-term efficacy results, the safety profile of pramipexole proved to be a significant clinical challenge. A serious problem in the study group was the phenomenon of phase switching to a manic or hypomanic episode (switching), the symptoms and adverse events of which were reported in as many as 44% of patients treated with pramipexole, compared to 29% in the placebo group (the authors noted that most phase switches in the placebo group occurred in patients without antipsychotic medication; the risk of phase switching was further exacerbated by the fact that 56% of the study participants were taking classic antidepressants). Furthermore, one of the patients on pramipexole experienced a full, severe relapse of mania with psychotic symptoms, which resulted in hospitalization. It was also noted that patients in the pramipexole group more frequently reported adverse events related to difficulties in impulse control (e.g., gambling, excessive shopping, or eating), which affected 33% of them [55].

### 3.4. Modafinil

Modafinil is a stimulant drug, primarily used to treat excessive sleepiness associated with narcolepsy, sleep apnea, and shift-work sleep disorder. Unlike traditional stimulants, it has minimal addictive potential. This substance affects numerous neurotransmitter systems in the brain, including increasing the release of catecholamines, serotonin, and glutamate, activating orexin and histamine neurons, and reducing GABA secretion. However, research indicates that the only structures modafinil directly binds to are the dopamine transporters (DAT) and norepinephrine transporters (NET). Research indicates that the dopaminergic system plays a key role in the drug’s effects and that close cooperation between the D1 and D2 dopamine receptors is necessary to induce wakefulness, with the D2 receptor playing a primary role. The effect of modafinil on other neurotransmitter systems appears to be merely secondary to its effects on dopamine and noradrenaline [56]. More recent studies are shifting the emphasis from the receptors themselves to brain circuits and networks, suggesting that modafinil’s action may also be understood as modulation of corticocerebellar connectivity, involving a direct enhancement of communication between cerebellar structures and the prefrontal cortex. Crucially, this reorganized neural network activity precisely aligns with the spatial distribution of dopamine (D2) and histamine (H3) receptors. This means that these receptors act as final “switches” in these circuits, translating the drug’s chemical effects into tangible improvements in concentration, executive functions, and the regulation of wakefulness [57]. Studies on depression with inadequate response to treatment in bipolar disorder may provide important data on modafinil’s effectiveness in potentially overcoming treatment resistance. One clinical trial evaluated this medication as an adjunct therapy in patients whose symptoms were insufficiently responsive to standard mood stabilizer therapy (either alone or in combination with antidepressants). The six-week, double-blind, placebo-controlled study included 85 patients aged 18–65. Treatment efficacy was assessed primarily by analyzing changes in the Inventory of Depressive Symptoms (IDS) score. It demonstrated that patients taking modafinil (at an average dose of 177 mg/day) achieved significantly greater improvement in depressive symptoms compared to the placebo group, with clinically significant effects observed as early as week 2 of therapy. The positive response rate was 44% in the modafinil group compared to 23% in the placebo group, while the complete remission rate was 39% and 18%, respectively. Modafinil had a good safety profile, with headache being the most common adverse event. Importantly, from a safety perspective, modafinil use did not significantly increase the risk of a phase transition to mania or hypomania (14.6%) compared to placebo (11.4%). These results suggest that modafinil at doses of 100–200 mg daily may be an effective and safe option for augmenting (strengthening) therapy in patients with difficult-to-treat depressive symptoms [58]. The effectiveness of this stimulant has been confirmed not only in clinical trials [58] but also in everyday psychiatric practice. This is illustrated by a case study from 2025 describing the use of modafinil. It involved a 74-year-old patient with long-standing bipolar disorder and TRBD, manifested by severe anhedonia, excessive sleepiness, and lack of motivation. Due to multiple comorbidities, including chronic kidney disease, cerebellar ataxia, and pulmonary fibrosis, the man was intolerant or unresponsive to traditional treatment with lithium, antidepressants, and multiple antipsychotics, and electroconvulsive therapy was also excluded for anesthetic reasons. Modafinil was added as augmentation therapy (venlafaxine 75 mg and lamotrigine 300 mg), gradually increasing the dose to 200 mg daily. After 16 weeks of therapy, improvement in mood, motivation, and alertness was observed. The patient’s MADRS score dropped from 29 to just 6 points, representing a 79% reduction in symptoms. Importantly, and consistent with the results of a previously reported study [58], the drug was well tolerated, did not cause any adverse effects, and did not destabilize mood toward mania [59].

### 3.5. Lamotrigine

Lamotrigine is an antiepileptic drug originally developed and introduced for the treatment of epilepsy. Its action is primarily associated with the inhibition of voltage-gated sodium channels (VGSCs), which reduces neuronal hyperexcitability and may also limit glutamate release [60]. Cutaneous adverse reactions are of particular importance in the safety profile, as, in addition to more common skin reactions such as rash, rare but severe forms such as Stevens-Johnson syndrome (SJS) and toxic epidermal necrolysis (TEN) can occur. In a review of 122 randomized trials, skin reactions were reported in 8.3% of patients, while the incidence of SJS/TEN was 0.04% [61]. Gradual dose titration is essential for the safe use of lamotrigine, as more rapid dose escalation is associated with an increased risk of cutaneous adverse events [62]. A multicenter, open-label, randomized trial (introduced in the section on risperidone), conducted as part of the extensive STEP-BD research program in 2006, assessed the efficacy of lamotrigine, inositol, or risperidone augmentation in patients with treatment-resistant bipolar depression. The study enrolled 66 adult patients diagnosed with bipolar I or II disorder whose ongoing depressive episode had failed to improve despite optimal doses of mood stabilizers combined with at least one antidepressant. The study utilized a specific equipoise randomization design, in which patients were randomly assigned only to medications for which they had previously consented. The treatment protocol for the lamotrigine arm involved adding lamotrigine to existing therapy and slowly titrating it (from 50 mg/day) to a target dose of 150–250 mg/day over a maximum period of 16 weeks. The primary endpoint was the rate of full recovery, defined as the presence of no more than two mood symptoms (defined as single symptoms meeting the DSM-IV threshold criteria for a major depressive episode, mania, or hypomania) and the absence of any significant symptoms for the full 8 weeks, which in practice meant a state fully consistent with the rigorous DSM-IV definition of complete remission. The results for this criterion did not show statistically significant differences between the pairs of studied drugs, but the rate of full recovery was highest for lamotrigine at 23.8%, compared to 17.4% for inositol and only 4.6% for risperidone. A significantly more pronounced advantage of lamotrigine was observed for secondary endpoints—patients treated with this medication had significantly lower depressive symptom severity, lower overall clinical severity, and significantly higher overall functioning at the end of the study compared to those receiving inositol or risperidone. Furthermore, as an indicator of better tolerability and efficacy, patients assigned to lamotrigine remained in the study significantly longer than those in the other groups. Despite methodological limitations, such as small sample size, lack of blinding (open-label), and reduced statistical power, the authors considered the lamotrigine results a clear signal of its clinical utility in refractory bipolar depression [33].

### 3.6. Celecoxib

Celecoxib, a selective COX-2 inhibitor, is being investigated in psychiatry as an adjunctive treatment for mood disorders, particularly in the context of the inflammatory hypothesis of bipolar disorder. Its potential effects are related to inhibition of prostaglandin synthesis and attenuation of the inflammatory response, which provides a biological rationale for its use in bipolar depression [63]. In a study of treatment-resistant bipolar depression, a beneficial effect of celecoxib augmentation was observed [64]. However, more recent summaries indicate that the overall evidence remains limited and does not clearly support its efficacy in bipolar disorder. Available short-term studies suggest good tolerability of the drug, although data on long-term safety are still lacking [65]. Recent publications on celecoxib in treatment-resistant bipolar depression have focused primarily on its use as an adjunct to escitalopram, which well reflects the clinical practice of adjunctive therapy in this patient group. In a 10-week, randomized, double-blind, placebo-controlled study, Halaris assessed the efficacy and safety of celecoxib adjunctive therapy in patients with treatment-resistant bipolar depression [64]. Resistance was defined as failure to achieve remission after at least two adequate pharmacotherapy trials over a minimum of 8 weeks. The cohort consisted of 47 patients who were added to established mood-stabilizing therapy (excluding lithium) with escitalopram at a target dose of 20 mg/day in combination with celecoxib at a dose of 400 mg/day (or placebo) for 8 weeks. The results demonstrated a clear advantage of pharmacological modulation of inflammation: the group receiving the COX-2 inhibitor had a significantly higher rate of treatment response (78% vs. 45%) and remission (63% vs. 10%) compared to the control group. Importantly, the addition of celecoxib not only intensified the antidepressant response but also accelerated it—a significant reduction in depressive and anxiety symptoms was observed after the first week of therapy. The treatment proved to be completely safe and well-tolerated; no significant laboratory abnormalities or coagulation abnormalities were recorded. This study demonstrates that modulating the proinflammatory phenotype with NSAIDs, such as celecoxib, is a promising and highly effective strategy for overcoming treatment resistance in depressive episodes in patients with bipolar disorder [64]. In an 8-week secondary analysis of a previous clinical trial, Shkundin assessed the impact of polymorphisms in the brain-derived neurotrophic factor (BDNF and BDNF-AS) genes on the efficacy of combined escitalopram and celecoxib therapy [66]. In a cohort of 41 adult patients (21–65 years old) with treatment-resistant bipolar depression, verified using the Maudsley Staging Method, the rs1519480, rs6265, and rs10835210 variants were assessed. Modulation of inflammation with a COX-2 inhibitor (celecoxib) has been shown to significantly increase response and remission rates compared to escitalopram monotherapy, but this effectiveness is strongly dependent on the patient’s genotype. For example, the highest response and remission rates with combination therapy were observed in non-carriers of the A allele for rs6265 and the G allele for rs1519480, as well as in carriers of the A allele for rs10835210. Although changes in serum BDNF protein concentrations did not reach statistical significance throughout the study, the results clearly demonstrate that the genetic profile associated with neuroplasticity plays a key role in determining susceptibility to adjunctive anti-inflammatory treatment, representing an important step towards personalized pharmacotherapy [66].

### 3.7. Memantine

Memantine is an adamantane derivative and an uncompetitive NMDA receptor antagonist with moderate affinity, fast kinetics, and strong voltage dependence. At therapeutic concentrations, it preferentially blocks pathologically stimulated extrasynaptic receptors, sparing physiological synaptic transmission. As an uncompetitive open-channel blocker with a fast off-rate, memantine distinguishes between different patterns of receptor activation. During normal brain function, synaptic receptors open for only milliseconds—too briefly for memantine to significantly block them, which preserves essential neurotransmission and cellular survival pathways. However, during prolonged pathological conditions, memantine effectively enters and blocks the persistently open channels of extrasynaptic receptors. This precision prevents toxic calcium influx and cell death without causing the severe side effects typical of other, non-selective NMDAR antagonists [67]. It also antagonizes 5-HT3 and nicotinic receptors [68]. Due to the phenomenon of partial entrapment in the ion channel and, among other things, an additional effect on 5-HT3 receptors, memantine demonstrates significantly better tolerability than ketamine, which binds exclusively in the deep part of the channel, causing its long-lasting blockade, called “trapping” [68]. Due to its mild safety profile, resulting from bypassing physiological transmission, the drug rarely causes serious side effects in humans at therapeutic doses [67]. Adverse effects typical of strong NMDA blockers (e.g., ataxia, motor stereotypies, or amnesia) are observed almost exclusively after administration of high, supratherapeutic doses in animal models, although tolerance can develop during treatment. Although memantine demonstrated significant antidepressant activity in preclinical studies and demonstrated synergistic activity with classic antidepressants, small clinical trials have not yet confirmed its efficacy in treating major depression in humans, and the available literature does not provide data to assess its significance in the treatment of bipolar disorder [68]. Unfortunately, there are no studies on memantine specifically in the context of TRBD treatment. The closest approach to this condition is a double-blind, randomized augmentation study conducted in 2012 among patients with bipolar depression who had not responded adequately to lamotrigine [69]. The study included 29 patients with bipolar disorder previously treated with lamotrigine at a minimum dose of 100 mg for a minimum of four weeks. The entire experiment lasted eight weeks, during which the memantine dose was increased by 5 mg per week to a maximum of 20 mg. This dose was maintained until the end of the eighth week. Analysis of individual treatment stages revealed a significant reduction in depression severity in the memantine group over the first four weeks, during the titration phase. By the end of the fourth week, 57% of patients in the memantine group had a positive response to treatment (defined as a >50% decrease in HDRS symptoms), whereas only 20% of those in the placebo group had such a response. However, the primary analysis did not demonstrate a statistically significant advantage of memantine over placebo in reducing depressive symptoms (as measured by the HDRS) after the full eight weeks of augmentation therapy. The fact that these antidepressant effects were significant at four weeks but were lost by the eighth week suggests that current titration or dosing protocols may be insufficient for maintaining long-term remission. Memantine was well-tolerated, with side effects of mild severity and occurring with similar frequency in both groups. No patients withdrew from the study due to adverse events. No prolonged episodes of hypomania were observed among patients in the memantine group [69].

## 4. Discussion

Well-planned and justified polypharmacy is a purposeful approach in which each component of treatment must fulfill a precisely defined and well-defined function. Complex treatment regimens should be based on complementary mechanisms of drug action, where the pharmacodynamic whole exceeds the sum of the individual components, which should complement each other in terms of their effects [23]. In the practice of treating refractory bipolar disorder, this comes down to combining medications that affect various pathophysiological levels, from monoaminergic systems to the glutamatergic system. Adopting such a model is becoming a clinical necessity, as standard monotherapy rarely leads to complete remission. Given the limited effectiveness of simple regimens, rational augmentation strategies currently form the basis of treatment aimed at overcoming drug resistance [24]. Unfortunately, current reviews indicate that current pharmacological options for the treatment of bipolar depression are limited, and the disease itself does not respond satisfactorily to classical antidepressants (according to the classification system) [25]. For this reason, alternative strategies are increasingly being used in research and daily clinical practice, such as adding second-generation antipsychotics to mood stabilizers [23,29,33,37,43]. The antipsychotic drugs included in the review of TRBD therapy were second-generation neuroleptics such as aripiprazole [29], risperidone [33], cariprazine [37], and lurasidone [43]. Limitations of all neuroleptic trials included small populations and relatively high patient dropout rates due to adverse effects, which reached 70.6% for cariprazine and 68.3% for lurasidone, respectively [37,43]. The rate of clinical benefit, defined as a reduction in depressive symptoms or remission, remained relatively low. The most promising clinical response data were provided by studies of combination therapy with cariprazine; clinical efficacy in a 4-week follow-up was 45.1% [37]. Further observations, however, indicate that cariprazine used as adjunctive therapy may be a useful short-term strategy, as in the 24-week long-term perspective it loses its advantage (reduction in depressive symptoms by only an additional 15%) and is burdened with mass drug discontinuation due to lack of efficacy, deterioration of the patient’s condition, or the occurrence of adverse effects [38]. This data illustrate the essence of the problem with second-generation neuroleptic augmentation: the severe burden of adverse effects frequently outweighs their moderate antidepressant efficacy. This forces a change in approach—leaving the monoaminergic system and leaning towards solutions other than basic neurotransmitter pathways thanks to understanding the nature and biology of the disease.

Leaving aside strategies focusing on the monoaminergic system, intervention in the glutamatergic system should be considered [10]. A noteworthy therapeutic option in this regard is ketamine [44]. Its antidepressant effect develops relatively quickly. Unlike traditional antidepressants, which take several weeks to manifest, ketamine can induce significant clinical improvement within just a few hours of administration [45,48]. A study evaluating intravenous infusions of this drug in TRBD patients achieved rapid and significant clinical improvement, with (unlike multi-week therapies) a zero-dropout rate. The therapy was generally well tolerated, and the reported side effects (mostly mild) were transient. Importantly, from the safety perspective of this group of patients, the treatment did not induce a phase transition to a manic episode in any of the patients [49]. An older study with a smaller study population demonstrated a surprisingly rapid antidepressant effect of ketamine, visible within 40 min of starting the intravenous infusion [48]. Therefore, a significant advantage of ketamine undoubtedly includes the low risk of phase transition in patients with bipolar disorder [47]. Another drug affecting the glutamatergic system is memantine [67]. Although studies specifically assessing the efficacy of memantine in patients with TRBD are lacking, an 8-week augmentation study in patients with incomplete response to lamotrigine demonstrated its early antidepressant effect. After 4 weeks of therapy, the clinical response rate was significantly higher in the memantine group (57% vs. 20% for placebo), although this statistical advantage in symptom reduction was not maintained throughout the study. Despite the lack of long-term advantage over placebo, the intervention was characterized by excellent tolerability and safety, neither leading to treatment discontinuation nor inducing prolonged episodes of hypomania [69]. Analysis of studies on two drugs affecting the same system allows us to conclude that ketamine is excellent for treating depressive episodes requiring rapid intervention and control of clinical symptoms. However, the search for an oral alternative acting on NMDA receptors in the form of memantine requires optimization of dosage and duration of testing in longer clinical trials.

In contrast to antipsychotics, there are drugs that directly agonize dopaminergic receptors, e.g., pramipexole [51]. Although a 2004 study estimated the occurrence of a manic switch at 8.3% in the pramipexole group [54], more recent studies shed less favorable light on this statistic. The use of pramipexole as a long-term augmentation strategy in patients with treatment-resistant bipolar depression demonstrated a significant advantage over placebo in achieving clinical response (46%) and complete remission (31%) in a recent study. However, the promising efficacy of this drug may be drastically limited by its problematic safety profile, characterized by an alarming risk of inducing a phase switch to (hypo)mania (in as many as 44% of subjects) and frequent occurrence of impulse control disorders (33% of subjects) [55]. Comparing dopaminergic strategies with the previously mentioned glutamatergic interventions highlights a massive contrast in safety profiles. Despite the promising, long-lasting antidepressant effect of pramipexole, its 44% phase-switching rate creates a critical clinical limitation. In stark contrast, intravenous ketamine demonstrated a much safer profile, with a phase-switching rate of only 2.4%. This stark difference shifts the scale of clinical utility heavily towards strategies targeting the glutamatergic system. In practice, this may mean that a strong dopaminergic strategy may be relegated to a reserve role or, in extremely resistant cases, when clinical benefit is desired, even at the risk of adverse effects (switching). Another approach to augmentation therapy was the use of celecoxib, a COX-2 inhibitor [63]. Modulation of proinflammatory processes with celecoxib, used as an augmentation drug to escitalopram, is an effective and safe strategy for overcoming drug resistance in bipolar depression. In a placebo-controlled study, this intervention not only increased the clinical response rate (78% vs. 45%) and complete remission (63% vs. 10%) but also significantly accelerated symptom reduction, demonstrating very good tolerability [64]. However, the effectiveness of augmenting escitalopram with celecoxib in treatment-resistant bipolar depression, according to recent studies, may be dependent on the patient’s genetic profile. The study demonstrated that specific polymorphisms of neurotrophic factor genes (BDNF and BDNF-AS) were associated with different clinical responses and remission rates after the use of this adjunctive anti-inflammatory treatment [66]. This finding sheds new light on the pathogenesis of drug resistance among patients, which may be related to an unfavorable genetic profile and the underlying inflammatory process. To translate these findings into practice, biomarkers such as the Systemic Immune-Inflammation Index (SII) and BDNF polymorphisms must be explicitly mapped to personalized therapeutic outcomes. In clinical decision-making, identifying an elevated SII or specific BDNF risk variants could serve as a direct indicator to prioritize targeted anti-inflammatory augmentation, such as celecoxib. By integrating these genetic and inflammatory markers, clinicians can bypass standard trial-and-error approaches and implement personalized protocols for patients with TRBD, maximizing the chance of rapid remission.

## 5. Limitations

It is important to acknowledge several methodological limitations within the current body of evidence. Notably, the lack of large-scale randomized controlled trials (RCTs) and the reliance on studies with small sample sizes for several augmentation strategies—specifically highlighting aripiprazole and memantine—inherently limit the overall strength of our clinical recommendations. While these interventions show mechanistic promise, future extensive, double-blind RCTs are required to establish more robust and definitive treatment guidelines for TRBD.

## 6. Conclusions

Drug-resistant bipolar depression (TRBD) requires a departure from classical monotherapy in favor of precise polypharmacotherapy, targeting the glutamatergic system, dopaminergic system and neuroinflammatory phenomena. Among the available options, intravenous administration of ketamine may currently be the most effective rescue strategy with an unprecedentedly fast action, surpassing the usefulness profile of atypical antipsychotics or pramipexole, which are burdened with numerous side effects. Optimization of treatment in TRBD, including the use of anti-inflammatory therapies (e.g., celecoxib), should be based on personalization and biomarkers in the future. Due to the complex biology of the disease, the key challenge remains to conduct large, standardized clinical trials that will objectively verify the effectiveness of new active substances or existing but used off-label.

Table S1 is included in the Supplementary Material and shows a summary of research on selected augmentation strategies and polytherapy in TRBD.

## Figures and Tables

**Table 1:** ISBD criteria for adequate treatment trials prior to TRBD diagnosis [2].

Bipolar Disorder Subtype	Approved Pharmacological Interventions	Dose and Duration
Bipolar I Disorder (BD-I)	Quetiapine	300–600 mg/day for ≥8 weeks
	Lurasidone	20–120 mg/day for ≥6 weeks
	Cariprazine	1.5–3 mg/day for ≥6 weeks
	Lumateperone	42 mg/day for ≥6 weeks
	Olanzapine-fluoxetine combination	Olanzapine (6–12 mg/day) + Fluoxetine (25–75 mg/day) for ≥8 weeks
Bipolar II Disorder (BD-II)	Quetiapine	300–600 mg/day for ≥8 weeks
	Lumateperone	42 mg/day for ≥6 weeks

**Table 2:** Targeted adjunctive strategies for the management of TRBD.

Drug	Efficacy	Side Effects
Aripiprazole	Moderate clinical support for TRBD. Clinical response achieved in 27% and 33.3% of patients in small, uncontrolled trials [28,29]. A randomized study showed no significant advantage over placebo [30].	Akathisia, insomnia, anxiety, agitation, nausea, gastrointestinal symptoms, mild extrapyramidal symptoms. Discontinuation due to adverse events was common
Risperidone	Limited efficacy in TRBD; recovery rate was only 4.6% compared to higher rates for inositol and lamotrigine [31,32,33].	Extrapyramidal symptoms, hyperprolactinemia, sedation, weight gain, orthostatic hypotension
Cariprazine	In a retrospective study, 23.5% achieved response and 21.6% remission after 4 weeks [34,35,36,37]. Long-term study showed 40% HDRS reduction at 4 weeks, but only moderate improvement after 24 weeks [38].	Akathisia, extrapyramidal symptoms, nausea, insomnia, inner restlessness, somnolence, and tremor
Lurasidone	Significant symptomatic improvement in some; 33.3% clinical response rate but low remission rate (3.3%) at short-term follow-up [39,40,41,42,43].	Akathisia, somnolence, nausea, insomnia, tremor, weight gain, internal tension, and muscle rigidity
Ketamine i.v	Rapid antidepressant and anxiolytic effects within hours. In total, 79% clinical response and 36% remission in one study [44,45,46,47,48]. Another study showed significant improvement starting from the second week [49]. In addition, ketamine is characterized by a low risk of manic shift.	Dissociative symptoms, transient increase in blood pressure, dizziness, nausea, somnolence, and transient cognitive impairment
Pramipexole	Strong long-term results: 46% response and 31% remission in follow-up up to 48 weeks. Primary endpoint at 12 weeks was not statistically significant due to small sample size [50,51,52,53,54,55].	Somnolence, sudden sleep attacks, impulse control disorders, and a high rate of phase switching
Modafinil	44% response rate and 39% remission rate [56,57,58]. Case study showed 79% symptom reduction [59]. Effective for anhedonia and lack of motivation.	Headache was most common
Lamotrigine	Highest recovery rate (23.8%) compared to inositol and risperidone in STEP-BD. Significant improvement in functioning and lower clinical severity. [33,60,61,62]	Rash, rare severe reactions like Stevens-Johnson syndrome or toxic epidermal necrolysis (gradual dose titration is important for the safe use of lamotrigine)
Celecoxib	Significantly higher response (78% vs. 45%) and remission (63% vs. 10%) as adjunct to escitalopram [63,64,65,66]. Effectiveness may be dependent on genetic profile (BDNF)	Well-tolerated in short-term studies; no significant laboratory or coagulation abnormalities recorded
Memantine	Significant reduction in depression (57% response) after 4 weeks of titration, but advantage over placebo was not maintained at 8 weeks [67,68,69].	Somnolence, shakiness, blurred vision, headache, sharper sense of smell, difficulty concentrating, chest pain, increased libido

## Data Availability

The original contributions presented in this study are included in the article. Further inquiries can be directed to the corresponding author.

## Supplementary Materials

The following supporting information can be downloaded at https://www.mdpi.com/article/10.3390/biomedicines14061185/s1. Table S1: Summary of research on selected augmentation strategies and polytherapy in TRBD.

## Abbreviations

The following abbreviations are used in this manuscript:

5-HTT	serotonin transporter
ACC	anterior cingulate cortex
AMPA	aminomethylphosphonic acid
BDNF	brain-derived neurotrophic factor
CINP	International College of Neuro-Psychopharmacology
COX-2	cyclooxygenase-2
CRP	c-reactive protein
DAT	dopamine transporter
GSK-3β	glycogen synthase kinase-3 beta
HAM-A	Hamilton Anxiety Rating Scale
HAMD-17	17-item Hamilton Depression Rating Scale
HCN	hyperpolarization-activated cyclic nucleotide-gated channels
HDRS	Hamilton Depression Rating Scale
IL	interleukin
ISBD	International Society for Bipolar Disorders
MADRS	Montgomery-Åsberg Depression Rating Scale
MHPG	3-Methoxy-4-hydroxyphenylglycol
NET	norepinephrine transporter
NF-κB	nuclear factor kappa-light-chain-enhancer of activated B cells
NMDA	N-methyl-D-aspartate
NOS	Not Otherwise Specified
PKC	protein kinase C
QIDS-SR	Quick Inventory of Depressive Symptomatology
SARI	serotonin antagonist and reuptake Inhibitor
SJS	Stevens–Johnson syndrome
SNRI	serotonin norepinephrine reuptake inhibitor
SSRI	selective serotonin reuptake Inhibitors
TCA	tricyclic antidepressants
TEN	toxic epidermal necrolysis
TNF-⍺	tumor necrosis factor ⍺
TPH2	tryptophan hydroxylase 2
TRBD	treatment-resistant bipolar depression
TrkB	tropomyosin receptor kinase B
VEGF	vascular endothelial growth factor
VGCC	voltage-gated calcium channel
VGluT1	vesicular glutamate transporter 1
VGSC	voltage-gated sodium channels
VPFC	ventral prefrontal cortex
WHO	World Health Organization
YMRS	Young Mania Rating Scale

## References

[1] Diaz A.P., Fernandes B.S., Quevedo J., Sanches M., Soares J.C. (2022). Treatment-resistant bipolar depression: Concepts and challenges for novel interventions. Braz. J. Psychiatry.

[2] Vieta E., McIntyre R.S., Suppes T., Van Rheenen T.E., Singh B., Miskowiak K.W., Young A.H., Yatham L.N., Ha K., Berk M. (2025). Defining Treatment-Resistant Bipolar Depression: Recommendations from the ISBD Task Force. Bipolar Disord..

[3] Nugent A.C., Bain E.E., Carlson P.J., Neumeister A., Bonne O., Carson R.E., Eckelman W., Herscovitch P., Zarate C.A., Charney D.S. (2013). Reduced post-synaptic serotonin type 1A receptor binding in bipolar depression. Eur. Neuropsychopharmacol..

[4] Oquendo M.A., Hastings R.S., Huang Y., Simpson N., Ogden R.T., Hu X.-Z., Goldman D., Arango V., Van Heertum R.L., Mann J.J. (2007). Brain Serotonin Transporter Binding in Depressed Patients with Bipolar Disorder Using Positron Emission Tomography. Arch. Gen. Psychiatry.

[5] Ashok A.H., Marques T.R., Jauhar S., Nour M.M., Goodwin G.M., Young A.H., Howes O.D. (2017). The dopamine hypothesis of bipolar affective disorder: The state of the art and implications for treatment. Mol. Psychiatry.

[6] Maletic V., Raison C. (2014). Integrated Neurobiology of Bipolar Disorder. Front. Psychiatry.

[7] Kurita M., Nishino S., Numata Y., Okubo Y., Sato T. (2014). The Noradrenaline Metabolite MHPG Is a Candidate Biomarker from the Manic to the Remission State in Bipolar Disorder I: A Clinical Naturalistic Study. PLoS ONE.

[8] Kurita M. (2016). Noradrenaline plays a critical role in the switch to a manic episode and treatment of a depressive episode. Neuropsychiatr. Dis. Treat..

[9] Womer F.Y., Kalmar J.H., Wang F., Blumberg H.P. (2009). A ventral prefrontal-amygdala neural system in bipolar disorder: A view from neuroimaging research. Acta Neuropsychiatr..

[10] Ginsberg S.D., Hemby S.E., Smiley J.F. (2012). Expression profiling in neuropsychiatric disorders: Emphasis on glutamate receptors in bipolar disorder. Pharmacol. Biochem. Behav..

[11] Jones G.H., Vecera C.M., Pinjari O.F., Machado-Vieira R. (2021). Inflammatory signaling mechanisms in bipolar disorder. J. Biomed. Sci..

[12] Grande I., Fries G.R., Kunz M., Kapczinski F. (2010). The Role of BDNF as a Mediator of Neuroplasticity in Bipolar Disorder. Psychiatry Investig..

[13] Muneer A. (2017). Wnt and GSK3 Signaling Pathways in Bipolar Disorder: Clinical and Therapeutic Implications. Clin. Psychopharmacol. Neurosci..

[14] Saxena A., Scaini G., Bavaresco D.V., Leite C., Valvassoria S.S., Carvalho A.F., Quevedo J. (2017). Role of Protein Kinase C in Bipolar Disorder: A Review of the Current Literature. Complex Psychiatry.

[15] Machado-Vieira R., Courtes A.C., Zarate C.A., Henter I.D., Manji H.K. (2023). Non-canonical pathways in the pathophysiology and therapeutics of bipolar disorder. Front. Neurosci..

[16] Cyrino L.A.R., Delwing-de Lima D., Ullmann O.M., Maia T.P. (2021). Concepts of Neuroinflammation and Their Relationship with Impaired Mitochondrial Functions in Bipolar Disorder. Front. Behav. Neurosci..

[17] Wollenhaupt-Aguiar B., Kapczinski F., Pfaffenseller B. (2021). Biological Pathways Associated with Neuroprogression in Bipolar Disorder. Brain Sci..

[18] Fountoulakis K.N., Yatham L.N., Grunze H., Vieta E., Young A.H., Blier P., Tohen M., Kasper S., Moeller H.J. (2020). The CINP Guidelines on the Definition and Evidence-Based Interventions for Treatment-Resistant Bipolar Disorder. Int. J. Neuropsychopharmacol..

[19] Hidalgo-Mazzei D., Berk M., Cipriani A., Cleare A.J., Di Florio A., Dietch D., Geddes J.R., Goodwin G.M., Grunze H., Hayes J.F. (2019). Treatment-resistant and multi-therapy-resistant criteria for bipolar depression: Consensus definition. Br. J. Psychiatry.

[20] Decker K., Murata S., Baig N., Hasan S., Halaris A. (2023). Utilizing the Systemic Immune-Inflammation Index and Blood-Based Biomarkers in Association with Treatment Responsiveness amongst Patients with Treatment-Resistant Bipolar Depression. J. Pers. Med..

[21] Lally N., Nugent A.C., Luckenbaugh D.A., Ameli R., Roiser J.P., Zarate C.A. (2014). Anti-anhedonic effect of ketamine and its neural correlates in treatment-resistant bipolar depression. Transl. Psychiatry.

[22] Villaseñor A., Ramamoorthy A., dos Santos M.S., Lorenzo M.P., Laje G., Zarate C., Barbas C., Wainer I.W. (2014). A pilot study of plasma metabolomic patterns from patients treated with ketamine for bipolar depression: Evidence for a response-related difference in mitochondrial networks. Br. J. Pharmacol..

[23] Goldberg J.F. (2019). Complex Combination Pharmacotherapy for Bipolar Disorder: Knowing When Less Is More or More Is Better. Focus.

[24] Hui Poon S., Sim K.J., Baldessarini R. (2015). Pharmacological Approaches for Treatment-resistant Bipolar Disorder. Curr. Neuropharmacol..

[25] Yalin N., Young A.H. (2020). Pharmacological Treatment of Bipolar Depression: What are the Current and Emerging Options?. Neuropsychiatr. Dis. Treat..

[26] Tuplin E.W., Holahan M.R. (2017). Aripiprazole, A Drug that Displays Partial Agonism and Functional Selectivity. Curr. Neuropharmacol..

[27] Citrome L. (2006). A review of aripiprazole in the treatment of patients with schizophrenia or bipolar I disorder. Neuropsychiatr. Dis. Treat..

[28] Ketter T.A., Wang P.W., Chandler R.A., Culver J.L., Alarcon A.M. (2006). Adjunctive Aripiprazole in Treatment-Resistant Bipolar Depression. Ann. Clin. Psychiatry.

[29] Kemp D.E., Gilmer W.S., Fleck J., Straus J.L., Dago P.L., Karaffa M. (2007). Aripiprazole augmentation in treatment-resistant bipolar depression: Early response and development of akathisia. Prog. Neuropsychopharmacol. Biol. Psychiatry.

[30] Quante A., Zeugmann S., Luborzewski A., Schommer N., Langosch J., Born C., Anghelescu I., Wolf J. (2010). Aripiprazole as adjunct to a mood stabilizer and citalopram in bipolar depression: A randomized placebo-controlled pilot study. Hum. Psychopharmacol..

[31] Leysen J.E., Janssen P.M., Megens A.A., Schotte A. (1994). Risperidone: A novel antipsychotic with balanced serotonin-dopamine antagonism, receptor occupancy profile, and pharmacologic activity. J. Clin. Psychiatry.

[32] Conley R.R. (2000). Risperidone side effects. J. Clin. Psychiatry.

[33] Nierenberg A.A., Ostacher M.J., Calabrese J.R., Ketter T.A., Marangell L.B., Miklowitz D.J., Miyahara S., Bauer M.S., Thase M.E., Wisniewski S.R. (2006). Treatment-resistant bipolar depression: A STEP-BD equipoise randomized effectiveness trial of antidepressant augmentation with lamotrigine, inositol, or risperidone. Am. J. Psychiatry.

[34] Calabrese F., Tarazi F.I., Racagni G., Riva M.A. (2020). The role of dopamine D3 receptors in the mechanism of action of cariprazine. CNS Spectr..

[35] Hope J., Keks N.A. (2022). Cariprazine: A new partial dopamine agonist with a familiar profile. Australas. Psychiatry Bull. R. Aust. N. Z. Coll. Psychiatr..

[36] Do A., Keramatian K., Schaffer A., Yatham L. (2021). Cariprazine in the Treatment of Bipolar Disorder: Within and Beyond Clinical Trials. Front. Psychiatry.

[37] Teobaldi E., Pessina E., Martini A., Cattaneo C.I., De Berardis D., Martiadis V., Maina G., Rosso G. (2024). Cariprazine Augmentation in Treatment-Resistant Bipolar Depression: Data from a Retrospective Observational Study. Curr. Neuropharmacol..

[38] Martiadis V., Pessina E., Martini A., Raffone F., Vignapiano A., De Berardis D. (2024). Cariprazine add-on in resistant bipolar depression. Long-term effectiveness and safety data from a multicentric real-world experience. Eur. Psychiatry.

[39] Siwek M., Krupa A.J., Wasik A. (2020). Lurasidone–pharmacodynamic and pharmacokinetic properties, clinical potential and interaction risk. Pharmacother. Psychiatry Neurol..

[40] Ishibashi T., Horisawa T., Tokuda K., Ishiyama T., Ogasa M., Tagashira R., Matsumoto K., Nishikawa H., Ueda Y., Toma S. (2010). Pharmacological profile of lurasidone, a novel antipsychotic agent with potent 5-hydroxytryptamine 7 (5-HT7) and 5-HT1A receptor activity. J. Pharmacol. Exp. Ther..

[41] Loebel A., Cucchiaro J., Silva R., Kroger H., Sarma K., Xu J., Calabrese J.R. (2014). Lurasidone as adjunctive therapy with lithium or valproate for the treatment of bipolar I depression: A randomized, double-blind, placebo-controlled study. Am. J. Psychiatry.

[42] Lin Y.-W., Chen Y.-C.B., Hung K.-C., Liang C.-S., Tseng P.-T., Carvalho A.F., Vieta E., Solmi M., Lai E.C.-C., Lin P.-Y. (2024). Efficacy and acceptability of lurasidone for bipolar depression: A systematic review and dose-response meta-analysis. BMJ Ment. Health.

[43] Porceddu G., Pessina E., Cattaneo C.I., Martiadis V., Bianca C., Maina G., Rosso G. (2026). Real-world outcomes of lurasidone augmentation for treatment-resistant bipolar depression: A retrospective observational analysis. Front. Psychiatry.

[44] Lavender E., Hirasawa-Fujita M., Domino E.F. (2020). Ketamine’s dose related multiple mechanisms of actions: Dissociative anesthetic to rapid antidepressant. Behav. Brain Res..

[45] Matveychuk D., Thomas R.K., Swainson J., Khullar A., MacKay M.-A., Baker G.B., Dursun S.M. (2020). Ketamine as an antidepressant: Overview of its mechanisms of action and potential predictive biomarkers. Ther. Adv. Psychopharmacol..

[46] Joseph B., Parsaik A.K., Ahmed A.T., Erwin P.J., Singh B. (2021). A Systematic Review on the Efficacy of Intravenous Racemic Ketamine for Bipolar Depression. J. Clin. Psychopharmacol..

[47] Nunez N.A., Joseph B., Kumar R., Douka I., Miola A., Prokop L.J., Mickey B.J., Singh B. (2023). An Update on the Efficacy of Single and Serial Intravenous Ketamine Infusions and Esketamine for Bipolar Depression: A Systematic Review and Meta-Analysis. Brain Sci..

[48] Zarate C.A., Brutsche N.E., Ibrahim L., Franco-Chaves J., Diazgranados N., Cravchik A., Selter J., Marquardt C.A., Liberty V., Luckenbaugh D.A. (2012). Replication of Ketamine’s Antidepressant Efficacy in Bipolar Depression: A Randomized Controlled Add-On Trial. Biol. Psychiatry.

[49] Cuomo A. (2025). Symptom modulation and tolerability of intravenous ketamine in treatment-resistant bipolar depression: A retrospective study. J. Affect. Disord..

[50] Constantinescu R. (2008). Update on the use of pramipexole in the treatment of Parkinson’s disease. Neuropsychiatr. Dis. Treat..

[51] Piercey M.F. (1998). Pharmacology of pramipexole, a dopamine D3-preferring agonist useful in treating Parkinson’s disease. Clin. Neuropharmacol..

[52] Dionys V., Sienaert P. (2021). Pramipexole in bipolar depression: A literature review and clinical recommendations. Tijdschr. Voor Psychiatr..

[53] Wilson S.M., Wurst M.G., Whatley M.F., Daniels R.N. (2020). Classics in Chemical Neuroscience: Pramipexole. ACS Chem. Neurosci..

[54] Goldberg J.F., Burdick K.E., Endick C.J. (2004). Preliminary randomized, double-blind, placebo-controlled trial of pramipexole added to mood stabilizers for treatment-resistant bipolar depression. Am. J. Psychiatry.

[55] McAllister-Williams R.H., Goudie N., Azim L., Bartle V., Berger M., Butcher C., Chadwick T., Clare E., Courtney P., Dixon L. (2025). A randomised double-blind, placebo-controlled trial of pramipexole in addition to mood stabilisers for patients with treatment-resistant bipolar depression (the PAX-BD study). J. Psychopharmacol..

[56] Qu W.-M., Huang Z.-L., Xu X.-H., Matsumoto N., Urade Y. (2008). Dopaminergic D1 and D2 Receptors Are Essential for the Arousal Effect of Modafinil. J. Neurosci..

[57] Pizzi S.D., Tomaiuolo F., Ferretti A., Bubbico G., Onofrj V., Della Penna S., Sestieri C., Sensi S.L. (2025). Modulation of Cerebellar-Cortical Connectivity Induced by Modafinil and Its Relationship with Receptor and Transporter Expression. Biol. Psychiatry Cogn. Neurosci. Neuroimaging.

[58] Frye M.A., Grunze H., Suppes T., McElroy S.L., Keck P.E., Walden J., Leverich G.S., Altshuler L.L., Nakelsky S., Hwang S. (2007). A Placebo-Controlled Evaluation of Adjunctive Modafinil in the Treatment of Bipolar Depression. Am. J. Psychiatry.

[59] Mbanusi A., Kanani M.-K. (2025). Challenges of Treatment-Resistant Bipolar Depression in the Elderly: A Case Study of Successful Modafinil Augmentation. Cureus.

[60] Costa B., Vale N. (2023). Understanding Lamotrigine’s Role in the CNS and Possible Future Evolution. Int. J. Mol. Sci..

[61] Bloom R., Amber K.T. (2017). Identifying the incidence of rash, Stevens-Johnson syndrome and toxic epidermal necrolysis in patients taking lamotrigine: A systematic review of 122 randomized controlled trials. An. Bras. Dermatol..

[62] Nakamura T., Tomita M., Hirota S., Matsunaga T., Uchimura N. (2022). Impact of Selected Initial Titration Schedules on Safety and Long-Term Effectiveness of Lamotrigine for the Treatment of Mood Disorders. J. Clin. Psychopharmacol..

[63] Sethi R., Gómez-Coronado N., Walker A.J., Robertson O.D., Agustini B., Berk M., Dodd S. (2019). Neurobiology and Therapeutic Potential of Cyclooxygenase-2 (COX-2) Inhibitors for Inflammation in Neuropsychiatric Disorders. Front. Psychiatry.

[64] Halaris A., Cantos A., Johnson K., Hakimi M., Sinacore J. (2020). Modulation of the inflammatory response benefits treatment-resistant bipolar depression: A randomized clinical trial. J. Affect. Disord..

[65] Gędek A., Szular Z., Antosik A.Z., Mierzejewski P., Dominiak M. (2023). Celecoxib for Mood Disorders: A Systematic Review and Meta-Analysis of Randomized Controlled Trials. J. Clin. Med..

[66] Shkundin A., Wheeler H.E., Sinacore J., Halaris A. (2025). BDNF/BDNF-AS Gene Polymorphisms Modulate Treatment Response and Remission in Bipolar Disorder: A Randomized Clinical Trial. J. Pers. Med..

[67] Xia P., Chen H.V., Zhang D., Lipton S.A. (2010). Memantine Preferentially Blocks Extrasynaptic over Synaptic NMDA Receptor Currents in Hippocampal Autapses. J. Neurosci..

[68] Parsons C., Rammes G., Danysz W. (2008). Pharmacodynamics of Memantine: An Update. Curr. Neuropharmacol..

[69] Anand A., Gunn A.D., Barkay G., Karne H.S., Nurnberger J.I., Mathew S.J., Ghosh S. (2012). Early antidepressant effect of memantine during augmentation of lamotrigine inadequate response in bipolar depression: A double-blind, randomized, placebo-controlled trial. Bipolar Disord..

