# Supplementary material for: Pharmacotherapeutic Options in Drug-Resistant Bipolar Depression: From Molecular Mechanisms to Rational Polypharmacotherapy
Source: Biomedicines. 2026 May 23;14(6):1185. doi: 10.3390/biomedicines14061185 (PMC13296849; doi:10.3390/biomedicines14061185)
Supplement: Supplementary file 1 [file biomedicines-14-01185-s001.zip › biomedicines-4322107-supplementary.pdf]

**Table S1**. Summary of research on selected augmentation strategies and polytherapy in TRBD

| Medicine | Study Type / TRBD Approval Status | Main results and statistical significance | Main Advantages/Disadvantages in TRBD |
|---|---|---|---|
| Aripiprazol | Augmentation studies (small, uncontrolled) [28,29]; One randomized controlled trial did not show a significant advantage over placebo [30]. No TRBD approval. | Clinical response in small studies: 27-33% [28,29]. Randomized Trial: No Statistically Significant Advantage Over Placebo in Reducing Symptoms of Depression [30]. | *Advantages*: Moderately favorable metabolic profile. *Disadvantages*: Limited efficacy in TRBD (no advantage over placebo in RCT), high rate of akathisia and extrapyramidal symptoms (up to 47% of withdrawals [28]), high percentage of withdrawals due to adverse events. |
| Risperidon | Augmentation research (STEP-BD) [33]. No TRBD approval. | Cure rate: 4.6% (significantly lower than lamotrigine and inositol) [33]. | *Advantages*: No significant ones. *Disadvantages:* Limited efficacy, extrapyramidal symptoms, hyperprolactinemia, sedation, weight gain. |
| Kariprazyn | Observational (retrospective) studies [37,38]. Approved for the treatment of depressive episodes in bipolar I disease in adults. No TRBD approval as an augment. | Short-term (4 weeks): 23.5% response, 21.6% remission; overall clinical benefit >45% [37]. Long-term (24 weeks): moderate further improvement (approx. 15%), high withdrawal rate (70%) [38]. Akathisia, restlessness, insomnia, tremor. | *Advantages:* Potential short-term efficacy, especially in reducing anxiety [37]. *Disadvantages:* High percentage of withdrawal in the long term, akathisia, extrapyramidal symptoms. |
| Lurasidon | Observational (retrospective) studies [43]. Approved for the treatment of depressive episodes in bipolar I and II disease in adults. No TRBD approval as an augment. | Short-term (4 weeks): 33.3% response, 3.3% remission [43]. Significant improvement in anxiety. | *Advantages:* Reduced symptoms of depression and anxiety, favorable metabolic profile. *Disadvantages:* Low full remission rate, akathisia, drowsiness, nausea, weight gain. |
| Ketamine i.v. | Randomized, double-blind, placebo-controlled trials [48]; observational studies [49]. Used "off-label" in TRBD, an approved form of nasal esketamine for TRD (non-bipolar). | Quick effect (hours); 79% response, 36% remission in RCT [48]. Significant improvement since 2 weeks in an observational study [49]. Low statistically significant difference in improvement. | *Advantages:* The fastest and most pronounced antidepressant effect, low manic conversion rate (<2.4%) [47]. *Disadvantages:* Transient dissociative symptoms, increased blood pressure, dizziness. Requires intravenous administration and monitoring. |
| Pramipeksol | Randomized, double-blind, placebo-controlled (PAX-BD) [55]. No TRBD approval. | 12 weeks: no statistically significant difference (p=0.087); longer follow-up (up | *Advantages*: Promising long-term results in response and remission [55]. *Disadvantages:* High risk of manic |

| | | | |
|---|---|---|---|
| | | to 48 weeks): 46% response, 31% remission (statistically significant) [55]. | conversion (44%) and impulse control disorders (33%) [55]. Risk of drowsiness/sudden sleep attacks. |
| Modafinil | Randomized, double-blind, placebo-controlled trials [58]; case study [59]. No TRBD approval. | 44% response, 39% remission in RCT (vs. placebo 23%/18%) [58]. A 79% reduction in symptoms in a case study [59]. Statistically significant improvement in depression. | *Advantages:* Effective in anhedonia and lack of motivation, good safety profile (low risk of mania/hypomania in the study) [58]. *Disadvantages:* The most common side effect is headache. |
| Lamotrygina | A randomized, open-label trial (STEP-BD) [63]. Approved as a mood stabilizer in bipolar disorder. Used "off-label" as an augmentation in TRBD. | The highest rate of complete cure (23.8%) among the investigational drugs (inositol, risperidone) in STEP-BD; better improvement of functioning [63]. Statistically significant also compared to other arms of the study. | *Advantages*: Improvement in symptoms of depression, good tolerance with gradual title, lower risk of manic conversion. *Disadvantages*: Risk of rash, including SJS/TEN (requires slow titleing). |
| Celekoksyb | Randomized, double-blind, placebo-controlled trials (escitalopram augmentation) [65]. No TRBD approval. | 78% response (vs. 45% placebo), 63% remission (vs. 10% placebo) [65]. Significantly higher response and remission. BDNF genotype-dependent efficacy [67]. | *Advantages*: Significantly increases response and remission rates, fast action, well tolerated [65]. *Disadvantages:* Study data mainly with escitalopram, efficacy dependence on genotype. |
| Memantyna | Randomized, double-blind, placebo-controlled (lamotrigine augmentation) [70]. No TRBD approval. | Early antidepressant effect (4 weeks): 57% response (vs. 20% placebo); no statistically significant advantage over placebo at 8 weeks. [70]. | *Advantages*: Good tolerance, safe profile, no induction of hypomanic episodes. *Disadvantages*: Lack of long-term efficacy (no statistically significant advantage after 8 weeks), no data specifically for TRBD |
